# Supplementary material for: Fragmentation modes and the evolution of life cycles
Source: PLoS Comput Biol. 2017 Nov 22;13(11):e1005860. doi: 10.1371/journal.pcbi.1005860 (PMC5718564; doi:10.1371/journal.pcbi.1005860)
Supplement: S1 Text — (PDF) [file pcbi.1005860.s001.pdf]

Supporting information: Fragmentation modes and the evolution of life cycles

Yuriy Pichugin[1*], Jorge Peña[1,2,†], Paul B. Rainey[1,3,4], Arne Traulsen[1]

1 Max Planck Institute for Evolutionary Biology, August-Thienemann-Str. 2, 24306 Plön, Germany

2 GEOMAR Helmholtz Centre for Ocean Research Kiel, Evolutionary Ecology of Marine Fishes, Düsternbrooker Weg 20, 24105 Kiel, Germany

3 Ecole Supérieure de Physique et de Chimie Industrielles de la Ville de Paris (ESPCI ParisTech), CNRS UMR 8231, PSL Research University, 75231 Paris Cedex 05, France

4 New Zealand Institute for Advanced Study, Massey University, Private Bag 102904, Auckland 0745, New Zealand

* Corresponding author

† Current address: Institute for Advanced Study in Toulouse, 21 allée de Brienne, 31015 Toulouse Cedex 6, France

Contents

A Mixed fragmentation modes **2**

B Mixed fragmentation modes are dominated **4**

C Mixing between 1+1 and 2+1 is dominated **6**

D Characteristic equation of a pure fragmentation mode **8**

E Fragmentation modes are dominated by binary splitting **9**

F Optimality maps for $n = 4$ **11**

G Costly fragmentation **12**

H With proportional costs, fragmentation modes are dominated by binary splitting **14**

A Mixed fragmentation modes

A mixed fragmentation mode assigns a probability q_κ to each possible fragmentation pattern (or partition) $\kappa \vdash 2, \ldots, \kappa \vdash n$, where n is the maximum group size. Such probabilities satisfy $\sum_{\kappa \vdash j} q_\kappa = 1$ for $j = 2, \ldots, n$, i.e., when growing from size $j-1$ to j one of the partitions $\kappa \vdash j$ (including staying together without splitting, $\kappa = j$) will certainly occur. Additionally, we impose $q_n = 0$ so that, when growing from size $n-1$ to size n, a group can no longer stay together and will necessarily fragment. It follows that a given life cycle or fragmentation mode can be represented by a set of vectors of the form

$$\mathbf{q} = \left\{ \underbrace{(q_2, q_{1+1})}_{\kappa \vdash 2}, \underbrace{(q_3, q_{2+1}, q_{1+1+1})}_{\kappa \vdash 3}, \ldots, \underbrace{(q_n, q_{n-1+1}, q_{n-2+2}, \ldots, q_{1+1+\ldots+1})}_{\kappa \vdash n} \right\}. \tag{1}$$

Pure life cycles are a particular case where splitting probabilities q_κ are either zero or one, so that only one fragmentation pattern with more than one offspring group occurs.

A mixed life cycle can be understood as a set of reactions. A number $n-1$ of reactions, of the type

$$X_i \xrightarrow{d_i} 0 \tag{2}$$

model the death of groups; these are independent of the fragmentation mode. An additional number of reactions, one per each non-zero element of the vector $\mathbf{q}$, models the birth of units and the growth or fragmentation of groups. These reactions are of the type

$$X_i \xrightarrow{ib_i q_\kappa} \sum_{j=1}^{i+1} \pi_j(\kappa) X_j, \tag{3}$$

whereby a group of size i turns into a group of size $i+1$ at rate ib_j, and then instantly divides with probability q_κ into offspring groups in a way described by fragmentation pattern $\kappa \vdash i+1$, where parts equal to ℓ appear a number $\pi_\ell(\kappa)$ of times. These reactions depend on the life cycle, which specifies the probabilities of fragmentation patterns. For instance, the reaction

$$X_3 \xrightarrow{3b_3 q_{2+1+1}} X_2 + 2X_1,$$

stipulates that groups of size 3, which grow to size 4 at rate $3b_3$, will split with probability q_{2+1+1} into one group of size 2 and two groups of size 1. The growth of a group without fragmentation is also incorporated in the set of reactions given by (3). For instance, the reaction

$$X_3 \xrightarrow{3b_3 q_4} X_4,$$

stipulates that groups of size 3, which grow to size 4 at rate $3b_3$, will not split with probability q_4.

The sets of reactions (2) and (3) give rise to the system of differential equations

$$\dot{x_i} = \sum_{j=1}^{n-1} \sum_{\kappa \vdash j+1} q_\kappa \pi_i(\kappa) j b_j x_j - ib_i x_i - d_i x_i,\ i = 1, 2, \ldots, n-1, \tag{4}$$

where x_i denotes the abundance of groups of size i. This linear system can be represented in matrix form as

$$\dot{\mathbf{x}} = \mathbf{A}\mathbf{x}, \tag{5}$$

where $\mathbf{x} = (x_1, x_2, \ldots, x_{n-1})$ is the vector of abundances of the groups of different size and $\mathbf{A}$ is a $(n-1) \times (n-1)$ matrix with elements given by

$$a_{i,j} = jb_j \sum_{\kappa \vdash j+1} q_\kappa \pi_i(\kappa) - \delta_{i,j}\left(ib_i + d_i\right), \tag{6}$$

where $\delta_{i,j}$ is the Kronecker delta. Since $\pi_i(\kappa) = 0$ for $\kappa \vdash j+1$ and $i > j+1$ (a partition of a number has no parts larger than the number), the entries of $\mathbf{A}$ below the subdiagonal are zero. As an example,

consider $n = 4$. The projection matrix for this case is given by

$$\mathbf{A} = \begin{pmatrix} b_1 \sum\limits_{\kappa \vdash 2} q_\kappa \pi_1(\kappa) - b_1 - d_1 & 2b_2 \sum\limits_{\kappa \vdash 3} q_\kappa \pi_1(\kappa) & 3b_3 \sum\limits_{\kappa \vdash 4} q_\kappa \pi_1(\kappa) \\ b_1 \sum\limits_{\kappa \vdash 2} q_\kappa \pi_2(\kappa) & 2b_2 \sum\limits_{\kappa \vdash 3} q_\kappa \pi_2(\kappa) - 2b_2 - d_2 & 3b_3 \sum\limits_{\kappa \vdash 4} q_\kappa \pi_2(\kappa) \\ 0 & 2b_2 \sum\limits_{\kappa \vdash 3} q_\kappa \pi_3(\kappa) & 3b_3 \sum\limits_{\kappa \vdash 4} q_\kappa \pi_3(\kappa) - 3b_3 - d_3 \end{pmatrix}. \quad (7)$$

B Mixed fragmentation modes are dominated

For any fitness landscapes, mixed fragmentation modes are dominated by at least one pure life cycle. In other words, the optimal life cycle is pure.

To prove this result, consider the set of partitions $\kappa \vdash j$ for a given j, fix the probabilities of fragmentation patterns $\nu \vdash i \neq j$ to arbitrary values, and focus attention on the function

$$\lambda_1^j : S_j \to \mathbb{R},$$

mapping probability distributions in the ζ_j-simplex $S_j \subset \mathbb{R}^{\zeta_j}$ (specifying the probabilities of all partitions $\kappa \vdash j$) to the dominant eigenvalue λ_1^j of the associated projection matrix $\mathbf{A}$. Our goal is to show that, for any j, λ_1^j is a quasiconvex function, i.e., that

$$\lambda_1^j(\eta \mathbf{x}_1 + (1-\eta)\mathbf{x}_2) \leq \max\left\{\lambda_1^j(\mathbf{x}_1), \lambda_1^j(\mathbf{x}_2)\right\}$$

holds for all $\mathbf{x}_1, \mathbf{x}_2 \in S_j$ and $\eta \in [0, 1]$. Quasiconvexity of λ_1^j implies that λ_1^j achieves its maximum at an extreme point of S_j, i.e., at a probability distribution that puts all of its mass in a single fragmentation pattern. Quasiconvexity of λ_1^j for all j then implies that the maximum growth rate λ_1 is achieved by a pure fragmentation mode, and that mixed fragmentation modes are dominated.

To show that λ_1^j is quasiconvex, we restrict the function to an arbitrary line and check quasiconvexity of the resulting scalar function [1, p. 99]. More precisely, we aim to show that the function

$$f(t) = \lambda_1^j(\mathbf{u} + t\mathbf{v}),$$

is quasiconvex in t for any $\mathbf{u} \in S_j$ and $\mathbf{v} \in \mathbb{R}^{\zeta_j}$ such that $\mathbf{u} + t\mathbf{v} \in S_j$. We hence need to verify that

$$f(\tau t_1 + (1-\tau)t_2) \leq \max\{f(t_1), f(t_2)\} \quad (8)$$

holds for $\tau \in [0, 1]$.

To show this, note that the function $f(t) = \lambda_1^j(\mathbf{u} + t\mathbf{v})$ is given implicitly by the largest root of the characteristic polynomial

$$p(\lambda) = \det\left(\mathbf{A} - \lambda\mathbf{I}\right), \tag{9}$$

where the probabilities of fragmentation specified by $\mathbf{u} + t\mathbf{v}$ appear in the $(j-1)$-th column of the projection matrix $\mathbf{A}$ (see Eqs. (6) and (7)).

The right hand side of Eq. (9) can be written using a Laplace expansion along the $(j-1)$-th column of $\mathbf{A} - \lambda\mathbf{I}$:

$$\det(\mathbf{A} - \lambda\mathbf{I}) = \sum_{i=0}^{n-1} (-1)^{i+j-1}(a_{i,j-1} - \delta_{i,j-1}\lambda)M_{i,j-1}, \tag{10}$$

where $\delta_{i,j-1}$ is the Kronecker delta and $M_{i,j-1}$ is the $(i, j-1)$ minor of $\mathbf{A}$, i.e., the determinant of the submatrix obtained from $\mathbf{A}$ by deleting the i-th row and $(j-1)$-th column. Each minor $M_{i,j-1}$ is independent of t because the only entries of $\mathbf{A}$ that depend on t appear in the $(j-1)$-th column. Moreover, each entry $a_{i,j-1}$ is either zero or a linear function of t. Hence, $p(\lambda)$ is a polynomial on λ with coefficients that are linear in t, i.e., of the form

$$p(\lambda) = \sum_{k=0}^{n-1} \left(\alpha_k + \beta_k t\right)\lambda^k, \tag{11}$$

for some α_k, β_k. Moreover, since the leading coefficient must be $(-1)^{n-1}$ (the matrix $\mathbf{A}$ is $(n-1) \times (n-1)$), it follows that $\alpha_{n-1} = (-1)^{n-1}$ and $\beta_{n-1} = 0$.

Denote by $p_\tau(\lambda)$, $p_1(\lambda)$, and $p_2(\lambda)$ the characteristic polynomials corresponding to, respectively, the probability distributions given by $\mathbf{u} + \left[\tau t_1 + (1-\tau)t_2\right]\mathbf{v}$, $\mathbf{u} + t_1\mathbf{v}$, and $\mathbf{u} + t_2\mathbf{v}$. From Eq. (11), these are given by

$$p_\tau(\lambda) = \sum_{k=0}^{n-1} \left(\alpha_k + \beta_k\left[\tau t_1 + (1-\tau)t_2\right]\right)\lambda^k = \sum_{k=0}^{n-1} \alpha_k\lambda^k + \left[\tau t_1 + (1-\tau)t_2\right]\sum_{k=0}^{n-1} \beta_k\lambda^k, \tag{12a}$$

$$p_1(\lambda) = \sum_{k=0}^{n-1} \left(\alpha_k + \beta_k t_1\right)\lambda^k = \sum_{k=0}^{n-1} \alpha_k\lambda^k + t_1\sum_{k=0}^{n-1} \beta_k\lambda^k, \tag{12b}$$

$$p_2(\lambda) = \sum_{k=0}^{n-1} \left(\alpha_k + \beta_k t_2\right)\lambda^k = \sum_{k=0}^{n-1} \alpha_k\lambda^k + t_2\sum_{k=0}^{n-1} \beta_k\lambda^k. \tag{12c}$$

Subtracting Eq. (12b) from Eq. (12a), and Eq. (12c) from Eq. (12a), we can write

$$p_\tau(\lambda) - p_1(\lambda) = (t_2 - t_1)(1-\tau)\sum_{k=0}^{n-1} \beta_k\lambda^k,$$

$$p_\tau(\lambda) - p_2(\lambda) = (t_1 - t_2)\tau\sum_{k=0}^{n-1} \beta_k\lambda^k.$$

Note that the signs of these differences are always different, i.e., either (i) $p_\tau(\lambda) - p_1(\lambda) \geq 0$ and $p_\tau(\lambda) - p_2(\lambda) \leq 0$, or (ii) $p_\tau(\lambda) - p_1(\lambda) \leq 0$ and $p_\tau(\lambda) - p_2(\lambda) \geq 0$. In the first case, we have $p_1(\lambda) \leq p_\tau(\lambda) \leq p_2(\lambda)$ and in the second we have $p_2(\lambda) \leq p_\tau(\lambda) \leq p_1(\lambda)$, i.e., for each λ, $p_\tau(\lambda)$ lies between $p_1(\lambda)$ and $p_2(\lambda)$, or, equivalently

$$p_\tau(\lambda) \leq \max\{p_1(\lambda), p_2(\lambda)\}, \tag{13}$$

for all λ. Since λ_1^j is the largest root of $p(\lambda)$, and since $p_\tau(\lambda)$, $p_1(\lambda)$, and $p_2(\lambda)$ all have the same sign in the limit when λ tends to infinity (their leading coefficients are all equal to $\alpha_{n-1} = (-1)^{n-1}$), condition (13) implies condition (8), thus proving our claim. See Fig. A for an illustration.

C Mixing between 1+1 and 2+1 is dominated

To show that the life cycle mixing between fragmentation modes 1+1 and 2+1 with probability q represented in vector form as

$$\mathbf{q} = \{(q_2, q_{1+1}), (q_3, q_{2+1}, q_{1+1+1})\} = \{(q, 1-q), (0, 1, 0)\} \tag{14}$$

is dominated, consider its growth rate λ_1^q as a function of q, as given by the solution of characteristic equation

$$\lambda_1^q = \frac{b_1(1-2q) - (d_1 + d_2) + \sqrt{(d_1 + d_2 - (1-2q)b_1)^2 + 4b_1(2qb_2 + (1-2q)d_2)}}{2}.$$

We have $\lambda_1^q(0) = \lambda_1^{1+1}$ and $\lambda_1^q(1) = \lambda_1^{2+1}$. A sufficient condition for q to be dominated by either 1+1 or 2+1 is then that $\lambda_1^q(q)$ is monotonic in q. To show that this is the case, note that the derivative of λ_1^q with respect to q is given by

$$\frac{d\lambda_1^q}{dq} = b_1\left(-1 + \frac{(2q-1)b_1 + 2b_2 + d_1 - d_2}{\sqrt{((2q-1)b_1 + d_1 + d_2)^2 + 4b_1(2qb_2 - (2q-1)d_2) - 4d_1d_2}}\right),$$

and that such expression is equal to zero if and only if

$$b_1 - b_2 = d_1 - d_2 \tag{15}$$

which is independent of q. It follows that λ_1^q is either nonincreasing or nondecreasing in q, and hence that it attains its maximum at either $q = 0$, $q = 1$, or (when (15) is satisfied) at any $q \in [0, 1]$. Hence, q is dominated by either 1+1 or 2+1.

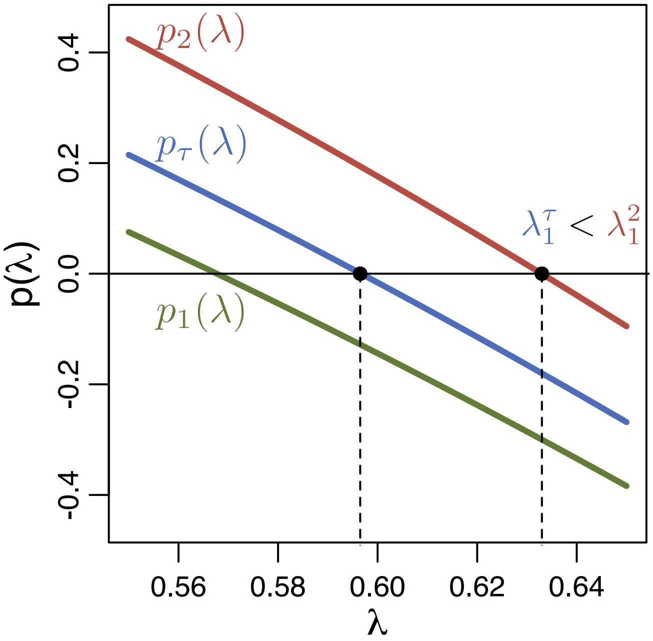

Figure A: **Population growth rate λ_1 is quasiconvex.** Consider two fragmentation modes $\mathbf{q}_1$ and $\mathbf{q}_2$ which differ only in the probabilities of fragmentation patterns at a single size j. Then, for any $0 \leq \tau \leq 1$ and corresponding fragmentation mode $\mathbf{q}_\tau = \tau\mathbf{q}_1 + (1-\tau)\mathbf{q}_2$, the polynomials $p(\lambda)$ given by Eq. (9) satisfy either $p_1(\lambda) \leq p_\tau(\lambda) \leq p_2(\lambda)$ or $p_2(\lambda) \leq p_\tau(\lambda) \leq p_1(\lambda)$. Thus, $\mathbf{q}_\tau$ leads to a lower growth rate than either $\mathbf{q}_1$ or $\mathbf{q}_2$, i.e., either $\lambda_1^\tau \leq \lambda_1^1$, or $\lambda_1^\tau \leq \lambda_1^2$ holds. Here, $j = 3$, $\mathbf{q}_1 = \{(0.9, 0.1), (\underline{0.5, 0.5, 0}), (0, 0, 0, 1, 0)\}$, $\mathbf{q}_2 = \{(0.9, 0.1), (\underline{0.5, 0, 0.5}), (0, 0, 0, 1, 0)\}$, and $\tau = 0.6$. The fitness landscape is given by $b_i = 1/i$, $d_i = 0$ for all i.

D Characteristic equation of a pure fragmentation mode

Consider the pure fragmentation mode $\kappa \vdash \ell$, whereby groups grow up to size ℓ and then fragment according to fragmentation pattern κ. The projection matrix is a $(\ell-1)\times(\ell-1)$ matrix of the form

$$\mathbf{A} = \begin{pmatrix} -b_1-d_1 & 0 & \cdots & 0 & (\ell-1)b_{\ell-1}\pi_1(\kappa) \\ b_1 & -2b_2-d_2 & 0 & \vdots & (\ell-1)b_{\ell-1}\pi_2(\kappa) \\ 0 & 2b_2 & -3b_3-d_3 & 0 & (\ell-1)b_{\ell-1}\pi_3(\kappa) \\ 0 & 0 & \ddots & \ddots & \vdots \\ 0 & 0 & \cdots & (\ell-2)b_{\ell-2} & (\ell-1)b_{\ell-1}\left(\pi_{\ell-1}(\kappa)-1\right)-d_{\ell-1} \end{pmatrix}.$$

The population growth rate is given by the leading eigenvalue λ_1 of $\mathbf{A}$, i.e., the largest solution of the characteristic equation

$$\det\left(\mathbf{A}-\lambda\mathbf{I}\right) = 0. \tag{16}$$

By using a Laplace expansion along the last column of $\mathbf{A}-\lambda\mathbf{I}$, we can rewrite the left hand side of the above expression (i.e., the characteristic polynomial of $\mathbf{A}$) as

$$\begin{aligned} \det\left(\mathbf{A}-\lambda\mathbf{I}\right) &= \sum_{i=1}^{\ell-2}(-1)^{i+\ell-1}(\ell-1)b_{\ell-1}\pi_i(\kappa)M_{i,\ell-1} \qquad (17)\\ &\quad + (-1)^{2(\ell-1)}\left[(\ell-1)b_{\ell-1}\pi_{\ell-1}(\kappa)-(\ell-1)b_{\ell-1}-d_{\ell-1}-\lambda\right]M_{\ell-1,\ell-1} \\ &= \sum_{i=1}^{\ell-1}(-1)^{i+\ell-1}(\ell-1)b_{\ell-1}\pi_i(\kappa)M_{i,\ell-1} - \left[(\ell-1)b_{\ell-1}+d_{\ell-1}+\lambda\right]M_{\ell-1,\ell-1} \qquad (18) \end{aligned}$$

where $M_{i,\ell-1}$ is the $(i,\ell-1)$-th minor of $\mathbf{A}-\lambda\mathbf{I}$. For all $i=1,\ldots,\ell-1$, the minor $M_{i,\ell-1}$ is the determinant of a block diagonal matrix, and hence equal to the product of the determinants of the diagonal blocks. Moreover, each diagonal block is either a lower triangular or an upper triangular matrix, whose determinant is given by the product of the elements in their main diagonals. We can then write

$$M_{i,\ell-1} = \prod_{j=1}^{i-1}\left(-jb_j-d_j-\lambda\right)\prod_{j=i}^{\ell-2}jb_j. \tag{19}$$

Substituting Eq. (19) into Eq. (18) and simplifying, we obtain

$$\begin{aligned}\det(\mathbf{A}-\lambda\mathbf{I}) &= (-1)^{\ell-2}\sum_{i=1}^{\ell-1}(\ell-1)b_{\ell-1}\pi_i(\kappa)\prod_{j=1}^{i-1}(jb_j+d_j+\lambda)\prod_{j=i}^{\ell-2}jb_j \\ &\quad -(-1)^{\ell-2}\left((\ell-1)b_{\ell-1}+d_{\ell-1}+\lambda\right)\prod_{j=1}^{\ell-2}(jb_j+d_j+\lambda) \\ &= (-1)^{\ell-2}\left[\prod_{j=1}^{\ell-1}jb_j\right]\left(\left[\sum_{i=1}^{\ell-1}\pi_i(\kappa)\prod_{j=1}^{i-1}\left(1+\frac{d_j+\lambda}{jb_j}\right)\right]-\prod_{j=1}^{\ell-1}\left(1+\frac{d_j+\lambda}{jb_j}\right)\right).\end{aligned}$$

Replacing this expression into the characteristic equation (16), dividing both sides by $(-1)^{\ell-1}\prod_{j=1}^{\ell-1}jb_j$, and simplifying, we finally obtain that the characteristic equation (16) can be written as

$$F_\ell(\lambda)-\sum_{i=1}^{\ell-1}\pi_i(\kappa)F_i(\lambda)=0, \tag{20}$$

where

$$F_i(\lambda)=\prod_{j=1}^{i-1}\left(1+\frac{d_j+\lambda}{jb_j}\right). \tag{21}$$

Note that the following two transformations:

$$\mathbf{d}\to\mathbf{d}-r, \qquad \lambda\to\lambda+r, \qquad r\le\min(\mathbf{d}),$$

and

$$\mathbf{d}\to s\mathbf{d}, \qquad \mathbf{b}\to s\mathbf{b}, \qquad \lambda\to s\lambda, \qquad s>0.$$

preserve the solution of Eq. (20) This allows us to set $b_1 = 1$ and $\min(\mathbf{d}) = 0$ without loss of generality.

E Fragmentation modes are dominated by binary splitting

We can show that, for any fitness landscape, binary fragmentation achieves a larger growth rate than splitting into more than two offspring groups. To prove this, consider (i) positive integers m, j, and k such that $m > j+k$, (ii) an arbitrary partition $\tau \vdash m-j-k$, and (iii) the following three fragmentation modes:

1. $\kappa_1 = j+k+\tau \vdash m$, whereby a complex of size m fragments into one complex of size j, one complex of size k, and a number of offspring complexes given by partition τ.

2. $\kappa_2 = (j+k) + \tau \vdash m$, whereby a complex of size m fragments into one complex of size $j+k$, and a number of offspring complexes given by partition τ.

3. $\kappa_3 = j + k \vdash (j+k)$, a binary splitting fragmentation mode whereby a complex of size $j+k$ fragments into two offspring complexes: one of size j, and one of size k.

Fragmentation mode κ_1 leads to a number of offspring groups equal to

$$n_1 = 2 + \sum_{\ell=1}^{m-j-k} \pi_\ell(\tau),$$

fragmentation mode κ_2 to a number of offspring groups equal to

$$n_2 = 1 + \sum_{\ell=1}^{m-j-k} \pi_\ell(\tau) = n_1 - 1,$$

and fragmentation mode κ_3 to a number of offspring groups equal to two. Denoting by λ_1^i the growth rate of fragmentation mode κ_i, we can show that, for any fitness landscape, either $\lambda_1^1 \leq \lambda_1^2$ or $\lambda_1^1 \leq \lambda_1^3$ holds, i.e., a fragmentation mode with more than two parts is dominated by either a fragmentation mode with one part less or by a fragmentation mode with exactly two parts. By induction, this implies that the optimal life cycle is always one within the class of binary fragmentation modes.

To prove that either $\lambda_1^1 \leq \lambda_1^2$ or $\lambda_1^1 \leq \lambda_1^3$ holds, let us denote by $p_i(\lambda)$ the characteristic polynomial associated to mode κ_i, as given by the left hand side of Eq. (20) after the replacement $\kappa = \kappa_i$. The growth rate λ_1^i of mode κ_i is hence the largest root of $p_i(\lambda)$. The polynomials $p_1(\lambda)$, $p_2(\lambda)$, and $p_3(\lambda)$ are then given by

$$p_1(\lambda) = F_m(\lambda) - \sum_{\ell=1}^{m-j-k} \pi_\ell(\tau) F_\ell(\lambda) - F_j(\lambda) - F_k(\lambda), \tag{22a}$$

$$p_2(\lambda) = F_m(\lambda) - \sum_{\ell=1}^{m-j-k} \pi_\ell(\tau) F_\ell(\lambda) - F_{j+k}(\lambda), \tag{22b}$$

$$p_3(\lambda) = F_{j+k}(\lambda) - F_j(\lambda) - F_k(\lambda). \tag{22c}$$

These polynomials satisfy the following two properties. First,

$$\lim_{\lambda \to \infty} p_i(\lambda) = \infty, \tag{23}$$

as the leading coefficient of the left hand side of Eq. (20) is always positive. Second, we can write

$$p_1(\lambda) = p_2(\lambda) + p_3(\lambda). \tag{24}$$

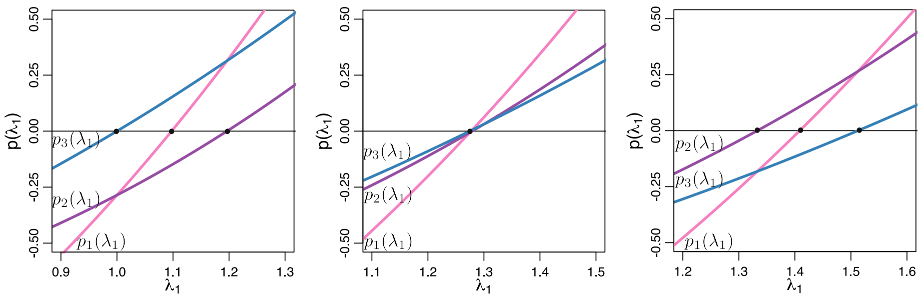

Figure B: **The population growth rate induced by a fragmentation mode with more than two offspring groups is dominated.** Consider the characteristic polynomials $p_i(\lambda_1)$ for partitions $\kappa_1 = 2+1+1$, $\kappa_2 = 3+1$ and $\kappa_3 = 2+1$. *Left*: Fitness landscape $\mathbf{b} = (1, 1, 1.4)$, $\mathbf{d} = (0, 0, 0)$. Since $p_2(\lambda_1^1) < 0$, κ_1 is dominated by κ_2 ($\lambda_1^1 < \lambda_1^2$ holds). *Center*: Fitness landscape $\mathbf{b} = (1, 2.6 - \sqrt{1.3}, 1.4)$, $\mathbf{d} = (0, 0, 0)$. Since $p_1(\lambda_1^1) = p_1(\lambda_1^2) = p_1(\lambda_1^3)$, κ_1 is weakly dominated by κ_2 ($\lambda_1^1 \leq \lambda_1^2$ holds). *Right*: Fitness landscape $\mathbf{b} = (1, 1.9, 1.4)$, $\mathbf{d} = (0, 0, 0)$. Since $p_3(\lambda_1^1) < 0$, κ_1 is dominated by κ_3 ($\lambda_1^1 < \lambda_1^3$ holds).

Now, evaluating Eq. (24) at λ_1^1, and since $p_1(\lambda_1^1) = 0$, it follows that $p_2(\lambda_1^1) = -p_3(\lambda_1^1)$. Hence, only one of the following three scenarios is satisfied: (i) $p_2(\lambda_1^1) < 0 < p_3(\lambda_1^1)$, (ii) $p_2(\lambda_1^1) = p_3(\lambda_1^1) = 0$, or (iii) $p_2(\lambda_1^1) > 0 > p_3(\lambda_1^1)$. If $p_2(\lambda_1^1) < 0 < p_3(\lambda_1^1)$, and by Eq. (23) and Bolzano's theorem, $\lambda_1^1 \leq \lambda_1^2$ holds. Likewise, if $p_2(\lambda_1^1) > 0 > p_3(\lambda_1^1)$, then $\lambda_1^1 \leq \lambda_1^3$ holds. Finally, if $p_2(\lambda_1^1) = p_3(\lambda_1^1) = 0$, then both $\lambda_1^1 \leq \lambda_1^2$ and $\lambda_1^1 \leq \lambda_1^3$ hold. See Fig. B for a graphical illustration of these arguments. We conclude that either $\lambda_1^1 \leq \lambda_1^2$ or $\lambda_1^1 \leq \lambda_1^3$ must hold, which proves our result.

F Optimality maps for $n = 4$

For $n = 4$ there are four pure fragmentation modes: 1+1, 2+1, 2+2, and 3+1. From Eq. (20), their characteristic polynomials are respectively given by

$$p_{1+1}(\lambda) = F_2(\lambda) - 2F_1(\lambda), \tag{25a}$$

$$p_{2+1}(\lambda) = F_3(\lambda) - F_2(\lambda) - F_1(\lambda), \tag{25b}$$

$$p_{2+2}(\lambda) = F_4(\lambda) - 2F_2(\lambda), \tag{25c}$$

$$p_{3+1}(\lambda) = F_4(\lambda) - F_3(\lambda) - F_1(\lambda). \tag{25d}$$

The optimality maps shown in Fig. 3 of the main text were obtained by comparing the largest

root of these characteristic polynomials, which we computed numerically. For fecundity landscapes, we tested fitness landscapes of the form $\{\mathbf{b}, \mathbf{d}\} = \{(1, b_2, b_3), (0, 0, 0)\}$ with b_2 and b_3 taken from a rectangular grid of size 300 by 300 with $b_2 \in [0, 5]$ and $b_3 \in [0, 5]$. For viability landscapes, we tested fitness landscapes of the form $\{\mathbf{b}, \mathbf{d}\} = \{(1, 1, 1), (5, d_2, d_3)\}$ with d_2 and d_3 taken from a rectangular grid of size 300 by 300 with $d_2 \in [0, 10]$ and $d_3 \in [0, 10]$.

The boundaries between areas of optimality can still be computed analytically. They are given by the fitness landscapes at which two fragmentation modes have the same population growth rate.

The following are the boundaries between areas of optimality under fecundity fitness landscape (assuming $b_1 = 1$ for simplicity):

- Between fragmentation modes 1+1 and 2+1: $b_2 = 1$, $b_3 < 1$.
- Between fragmentation modes 1+1 and 3+1: $b_3 = \frac{2}{3}\left(1 + \frac{1}{2b_2}\right)$, $b_2 < 1$.
- Between fragmentation modes 2+1 and 2+2: $b_3 = \frac{\zeta(2b_2+\zeta)}{3(2b_2-\zeta)}$, where $\zeta = \frac{\sqrt{1+8b_2}-1}{2}$, and $b_2 > 1$.
- Between fragmentation modes 3+1 and 2+2: $b_3 = \frac{2}{3}b_2\left(2b_2 - 1\right)\left(2 - \frac{1}{2b_2}\right)$ and $b_2 > 1$

The following are the boundaries between areas of optimality under viability fitness landscape (assuming $d_1 = 0$ for simplicity):

- Between fragmentation modes 1+1 and 2+1: $d_2 = 0$, $d_3 > 0$.
- Between fragmentation modes 1+1 and 3+1: $d_3 = \frac{3}{d_2+3} - 1$, $d_2 > 0$.
- Between fragmentation modes 2+1 and 2+2: $d_3 = 3\frac{2-d_2-\zeta}{2+d_2+\zeta} - \zeta$, where $\zeta = \frac{\sqrt{d_2^2-2d_2+9}-1-d_2}{2}$, and $d_2 < 0$.
- Between fragmentation modes 3+1 and 2+2: $d_3 = 3\frac{2-d_2-\zeta}{2+d_2+\zeta} - \zeta$, where $\zeta = \frac{\sqrt{d_2^2-6d_2+1}+1-d_2}{2}$ and $d_2 < 0$

G Costly fragmentation

For costly fragmentation, some cells are lost upon the fragmentation event. In this case the biological reactions are still given by Eqs. (2) and (3). However, under costly fragmentation the sum of sizes of offspring groups is smaller than the size of the parent group. Therefore, in Eq. (3), κ is a partition

of $i' \leq i+1$ (and not strictly of $i+1$ as it was under costless fragmentation). Indeed, $i' = i+1$ in the case of trivial partitions with one part (when a group grows without splitting), but $i' < i+1$ for nontrivial partitions with two or more parts (where the group grows in size by one cell and then splits). In this latter case, $i' = i - \pi + 2$ (where π is the number of offspring groups) for the case of proportional costs, and $i' = i$ for the case of fixed costs.

To illustrate the difference in the available sets of partitions for each of the three scenarios we investigate (costless fragmentation, fragmentation with proportional cost, fragmentation with fixed cost), consider the following possible reactions for a 4-cell group growing into a 5-cell group. For costless fragmentation, we have

$$
\begin{aligned}
&X_4 \xrightarrow{4b_4 q_5} X_5 && 5 \vdash 5 \text{ (no cell is lost)},\\
&X_4 \xrightarrow{4b_4 q_{4+1}} X_4 + X_1 && 4+1 \vdash 5 \text{ (no cell is lost)},\\
&X_4 \xrightarrow{4b_4 q_{3+2}} X_3 + X_2 && 3+2 \vdash 5 \text{ (no cell is lost)},\\
&X_4 \xrightarrow{4b_4 q_{3+1+1}} X_3 + 2X_1 && 3+1+1 \vdash 5 \text{ (no cell is lost)},\\
&X_4 \xrightarrow{4b_4 q_{2+2+1}} 2X_2 + X_1 && 2+2+1 \vdash 5 \text{ (no cell is lost)},\\
&X_4 \xrightarrow{4b_4 q_{2+1+1+1}} X_2 + 3X_1 && 2+1+1+1 \vdash 5 \text{ (no cell is lost)},\\
&X_4 \xrightarrow{4b_4 q_{1+1+1+1+1}} 5X_1 && 1+1+1+1+1 \vdash 5 \text{ (no cell is lost)}.
\end{aligned}
$$

For fragmentation with fixed cost, we have

$$
\begin{aligned}
&X_4 \xrightarrow{4b_4 q_5} X_5 && 5 \vdash 5 \text{ (no cell is lost)},\\
&X_4 \xrightarrow{4b_4 q_{3+1}} X_3 + X_1 && 3+1 \vdash 4 \text{ (1 cell is lost)},\\
&X_4 \xrightarrow{4b_4 q_{2+2}} 2X_2 && 2+2 \vdash 4 \text{ (1 cell is lost)},\\
&X_4 \xrightarrow{4b_4 q_{2+1+1}} X_2 + 2X_1 && 2+1+1 \vdash 4 \text{ (1 cell is lost)},\\
&X_4 \xrightarrow{4b_4 q_{1+1+1+1}} 4X_1 && 1+1+1+1 \vdash 4 \text{ (1 cell is lost)}.
\end{aligned}
$$

Finally, for fragmentation with proportional cost, we have

$$
\begin{aligned}
&X_4 \xrightarrow{4b_4 q_5} X_5 && 5 \vdash 5 \text{ (no cell is lost)},\\
&X_4 \xrightarrow{4b_4 q_{3+1}} X_3 + X_1 && 3+1 \vdash 4 \text{ (1 cell is lost)},\\
&X_4 \xrightarrow{4b_4 q_{2+2}} 2X_2 && 2+2 \vdash 4 \text{ (1 cell is lost)},\\
&X_4 \xrightarrow{4b_4 q_{1+1+1}} 3X_1 && 1+1+1 \vdash 3 \text{ (2 cells are lost)}.
\end{aligned}
$$

The combined probability of all outcomes of aggregate growth must be equal to one. In the case of costless fragmentation, this condition has been given by $\sum_{\kappa \vdash i+1} q_\kappa = 1$ for $i = 1, \ldots, n-1$. For costly fragmentation this condition changes to $\sum_{\kappa \vdash i'} q_\kappa = 1$ for $i = 1, \ldots, n-1$, with i' as defined above. The expressions for the system of differential equations and the projection matrix for general mixed strategies (Eqs. (4) and (7)) are changed accordingly. For pure fragmentation modes, the projection matrix given in the main text and the characteristic equation given in Eq. (20) remain valid, but κ is no longer a partition of $i+1$ but of i' as defined above.

H With proportional costs, fragmentation modes are dominated by binary splitting

For fragmentation with proportional costs, a group fragmenting into π offspring groups incurs a cost of $\pi - 1$ cells. In this case, similarly to the case for costless fragmentation, nonbinary fragmentation modes are dominated by binary fragmentation modes. To prove this, consider (i) positive integers m, j, and k such that $m > j + k + 4$, (ii) an arbitrary partition τ with $\pi \geq 2$ parts such that $\tau \vdash m - j - k - \pi - 2$, and (iii) the following three fragmentation modes:

1. $\kappa_1 = j + k + \tau \vdash m - \pi - 1$, whereby a complex of size m fragments into one complex of size j, one complex of size k, and π complexes given by partition τ, and $\pi + 1$ cells die.
2. $\kappa_2 = (j + k + 1) + \tau \vdash m - \pi$, whereby a complex of size m fragments into one complex of size $j + k + 1$ and π complexes given by partition τ, and π cells die.
3. $\kappa_3 = j + k \vdash (j + k)$, a binary fragmentation mode whereby a complex of size $j + k + 1$ fragments into two offspring complexes (one of size j and one of size k), and one cell dies.

Note that fragmentation mode κ_1 leads to $\pi + 2$ offspring groups, fragmentation mode κ_2 leads to $\pi + 1$ offspring groups, and fragmentation mode κ_3 leads to a number of offspring groups equal to two. The rest of the proof is analogous to the one given in Appendix E for the case of costless fragmentation and will be omitted.

References

[1] Stephen Boyd and Lieven Vandenberghe. *Convex optimization*. Cambridge university press, 2004.
